# EGFR phosphorylation of DCBLD2 recruits TRAF6 and stimulates AKT-promoted tumorigenesis

**DOI:** 10.1172/JCI194718

**Published:** 2025-05-15

**Authors:** Haizhong Feng, Giselle Y. Lopez, Chung Kwon Kim, Angel Alvarez, Christopher G. Duncan, Ryo Nishikawa, Motoo Nagane, An-Jey A. Su, Philip E. Auron, Matthew L. Hedberg, Lin Wang, Jeffery J. Raizer, John A. Kessler, Andrew T. Parsa, Wei-Qiang Gao, Sung-Hak Kim, Mutsuko Minata, Ichiro Nakano, Jennifer R. Grandis, Roger E. McLendon, Darell D. Bigner, Hui-Kuan Lin, Frank B. Furnari, Webster K. Cavenee, Bo Hu, Hai Yan, Shi-Yuan Cheng

Original citation: *J Clin Invest*. 2014;124(9):3741–3756. https://doi.org/10.1172/JCI73093

Citation for this retraction: *J Clin Invest*. 2025;135(10):e194718. https://doi.org/10.1172/JCI194718

Errors were recently identified in Figures 2E, 4A, 5B, and 5C, Supplemental Figure 4A, Supplemental Figure 14, Supplemental Figure 19, and the unedited blot images for Figure 5B. Although the authors have indicated that original source data support the findings in these figure panels, the corresponding authors are retracting the article due to the number of errors.

## Version 1 05/15/2025

Electronic publication

